# Light Stability, Pro-Apoptotic and Genotoxic Properties of Silver (I) Complexes of Metronidazole and 4-Hydroxymethylpyridine against Pancreatic Cancer Cells In Vitro

**DOI:** 10.3390/cancers12123848

**Published:** 2020-12-20

**Authors:** Dominik Żyro, Agnieszka Śliwińska, Izabela Szymczak-Pajor, Małgorzata Stręk, Justyn Ochocki

**Affiliations:** 1 Departament of Bioinorganic Chemistry, Chair of Medicinal Chemistry, Medical University of Łódź, Muszyńskiego 1, 90-151 Łódź, Poland; dominik.zyro@umed.lodz.pl (D.Ż.); justyn.ochocki@umed.lodz.pl (J.O.); 2 Department of Nucleic Acids Biochemistry, Medical University of Łódź, Pomorska 251, 92-213 Łódź, Poland; izabela.szymczak@umed.lodz.pl (I.S.-P.); malgorzata.strek@umed.lodz.pl (M.S.)

**Keywords:** cytotoxicity, apoptosis, genotoxicity, medicinal chemistry, silver (I), complex, metronidazole, 4-hydroxymethylpyridine, light stability, pancreatic cancer cells, UV-A rays

## Abstract

**Simple Summary:**

Antimicrobial properties of silver (I) ion and its complexes with metronidazole and 4-hydroxymethylpyridine are well recognized. However, little is known about its anticancer activity toward human pancreatic cancer cells. Our in vitro study revealed that silver (I) ion and its complexes with metronidazole and 4-hydroxymethylpyridine induced pancreatic cancer cells death associated with genotoxic and proapoptotic properties. In turn, the stability of active substances is of crucial importance because it determines the efficacy and applicability in clinical use. Therefore, we also evaluated photostability of silver (I) nitrate and its complexes with metronidazole and 4- hydroxymethylpyridine. Our results showed that studied complexes are more photochemically stable than silver salts, which makes them better candidates for clinical therapy.

**Abstract:**

Antimicrobial properties of silver (I) ion and its complexes are well recognized. However, recent studies suggest that both silver (I) ion and its complexes possess anticancer activity associated with oxidative stress-induced apoptosis of various cancer cells. In this study, we aimed to investigate whether silver nitrate and its complexes with metronidazole and 4-hydroxymethylpyridine exert anticancer action against human pancreatic cancer cell lines (PANC-1 and 1.2B4). In the study, we compared decomposition speed for silver complexes under the influence of daylight and UV-A (ultraviolet-A) rays. We employed the MTT (3-(4,5-dimethylthiazol-2-yl)-2,5-diphenyltetrazonium bromide) assay to evaluate the cytotoxicity and the alkaline comet assay to determine genotoxicity of silver nitrate and its complexes. Flow cytometry and the Annexin V-FITC/PI apoptosis detection kit were used to detect the apoptosis of human pancreatic cancer cells. We found a dose dependent decrease of both pancreatic cancer cell line viability after exposure to silver nitrate and its complexes. The flow cytometry analysis confirmed that cell death occurred mainly via apoptosis. We also documented that the studied compounds induced DNA damage. Metronidazole and 4-hydroxymethylpyridine alone did not significantly affect viability and level of DNA damage of pancreatic cancer cell lines. Complex compounds showed better stability than AgNO_3_, which decomposed slower than when exposed to light. UV-A significantly influences the speed of silver salt decomposition reaction. To conclude, obtained data demonstrated that silver nitrate and its complexes exerted anticancer action against human pancreatic cancer cells.

## 1. Introduction

Numerous studies have revealed that metal ions and their complexes have cytotoxic and genotoxic activity. These properties, especially of complex compounds with well know ligands, are still under extensive investigation as effective drugs for treating various disease, including cancers and those evoked by microorganisms. Our team performed numerous studies that reported not only cytotoxic and genotoxic potential of metal ions and their complexes toward normal and cancer cells but also greater stability of metal ion complexes, as compared to metal ion salts [1,2,3,4,5,6].

In medicine, silver and its compounds have been used as drugs for hundreds of years [7]. The most known are silver nitrate (Argenti nitras, Lapis), silver sulfadiazine (Dermazine^®^), and silver sulfathiazole (Argosulfan^®^). In addition, the silver preparations include colloidal silver drugs Collargol and Protargol. The pharmacological properties of silver preparations are determined by the specific biological activity of Ag(I) ions, which arise as a result of the dissociation of its compounds. One of the main pharmacological properties of the preparations is their antimicrobial activity. They are mainly used as antiseptics. In some cases, silver nitrate as such or in the form of concentrated solutions is used for cauterization, warts, and the like. It was also demonstrated that silver ions (silver nitrate) exerted cytotoxic and genotoxic properties against different human cells via oxidative stress production [8,9]. Recently, anticancer action of silver nitrates related to apoptosis induction was demonstrated in H-ras 5RP7 cells [10]. Studies have also shown the antitumor effects of silver complex compounds [11]. Silver, which is a transition metal, has the ability to form complex compounds. This has been the subject of research for many years. Many complex molecules can become potential therapeutic agents due to the unique biological effects of the silver (I) ion. It is extremely desirable that ligands, as structural parts of the silver (I) coordination complex, should exhibit proven clinical efficacy, as do metronidazole and 4-hydroxymethylpyridine. As a result of the action of the silver (I) ion and ligand, one should expect an additive synergy effect, and even hyper-additional synergism. [12,13,14].

The ligands that we used also play an important role in activity against living cells. Active imidazole derivatives have been sought out and investigated for many years. In the 1950s, metronidazole (MTZ) (2-(2-methyl-5-nitro-1H-imidazol-1-yl) ethanol) was synthesized in Rhone-Poulenc labs in France. It belongs to the pharmacological groups A01AB17; J01XD01; G01AF01; D06BX01; P01AB01 (Antimicrobials).

It is an antibacterial and antiprotozoal preparation of the nitroimidazole group active against *Trichomonas vaginalis*, *Entamoeba histolytica,* and lamblia. It shows a pronounced bactericidal effect against anaerobic bacteria (*Bacteroides spp.*, *Fusobacterium spp.*, *Eubacterium spp.*, and *Clostridium spp.*) [15]. The drug is inactive against aerobic bacteria and fungi. It is used to treat trichomoniasis, giardiasis, and amoebic dysentery. Moreover, it treats and prevents anaerobic infections, as well as severe mixed anaerobic and aerobic infections (in combination with the appropriate antibiotic combination).

Synthetic pyridine (Py) derivatives are used as medicinal products. The best known are nifedipine, amlodipine (treatment of angina), pinacidil (used in hypertension), isoniazid and phthivazide (treatment of tuberculosis), piroxicam (anti-inflammatory effect), and nicetamide (CNS stimulant). The piperidine ring is part of the painkiller drug trimeridine and the neuroleptic haloperidol. The group of pyridine compounds includes vitamins PP and B6. Pyridine and piperidine are structural fragments of the alkaloids nicotine, anabazine, lobeline, and coniine.

Complex compounds of silver and metronidazole (MTZ) and 4-hydroxymethylpyridine (4-OHMePy) have been presented in our earlier papers [9,16,17]. In our current research, we aimed to evaluate whether cytotoxicity of silver nitrate and its complexes may be a result of apoptosis. We also focus on the genotoxicity of these compounds, determine their stability after exposure to light, and pay attention to pH changes in aqueous solutions of the complex compounds in relation to the solutions of ligands and silver salts that we used in the experiments [18].

The cytotoxicity and genotoxicity of platinum and silver compounds against cancer cells, which was particularly the subject of our work presented here, is significant. We were inspired to start this study to expand our scientific horizons. We are continuing research on Ag(I) compounds and other Pd(II) and Ru(II) metals, which, as elements, combine at least three common denominators: cytotoxicity and genotoxicity, an antitumor effect, and location in one period side-by-side in the table of periodic chemical elements. 

In this paper, we present results for the antitumor activity of silver (I) complexes with pharmaceuticals, i.e., metronidazole and 4-hydroxymethylpyridine, against human pancreatic cancer cells.

## 2. Results

### 2.1. Synthesis of Metronidazole Complex with Silver Nitrate

**Scheme 1:**
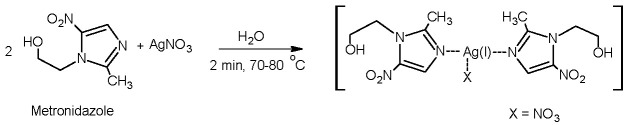
Synthesis of silver (I) complex of metronidazole.

The synthesis of the complex of the silver ion with metronidazole in the form of a nitrate salt was presented in our earlier papers [9,17]. The complex compound was synthesized in a simple one-step process by reacting AgNO_3_ with metronidazole (1:2) (Scheme 1) in water [9]. We confirmed the good reaction yield and purity of the resulting product (see Materials and Methods section).

### 2.2. Synthesis of 4-hydroxymethylpyridine Complex with Silver Nitrate

**Scheme 2:**
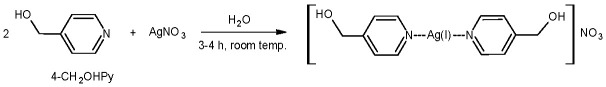
Synthesis of silver (I) complex of 4-hydroxymethylpyridine.

We previously described the synthesis of the silver ion complex compound with 4-hydroxymethylpyridine in the form of a nitrate salt [9,16]. Silver (I) 4-hydroxymethylpyridine complex was synthesized in an easy process by reacting AgNO_3_ with 4-hydroxymethylpyridine (1: 2) (Scheme 2) in water [9]. We confirmed good yield and purity of the resulting product (see Materials and Methods section).

### 2.3. Light Stability—Results of Physicochemical Tests

AgNO_3_ began to darken after 1 h of exposure on each of the groundworks. The complexes began to darken after 4 h on paper (other groundworks remained unchanged). Silver nitrate darkened completely after 24 h on all groundworks. The process of decomposition of the complexes proceeded quite quickly and the complexes darkened after about 3 days, decomposing the fastest on paper. On the glass, the distribution of ((4-OHMePy)_2_Ag)NO_3_ was observed (the remaining compounds remained unchanged). Blackout for ((4-OHMePy)_2_Ag)NO_3_ is visible on leather imitation (Figure S1 in the dark, Figure S2 in the light; see Supplementary Materials). The action of UV-A rays accelerates the process of decomposition of the silver complexes to a large extent; practically complete disintegration occurs after 3 min. (Figure S3 in UV-A; see Supplementary Materials). The complex of metronidazole with silver nitrate shows the best stability and resistance to UV-A radiation, while the complex of 4-hydroxymethylpyridine with silver nitrate is the least stable. Comparatively, the effect of light and UV-A radiation affects only the substances applied onto the surfaces which they can permeate, while the aqueous solutions are quite stable, and changes only occur after the natural evaporation of the solvent.

The measured pH values of the solutions of silver (I) complexes with metronidazole and 4-hydroxymethylpyridine and comparative solutions of ligands and salts (concentration of each 0.06 M) show that the synthesized compounds do not undergo hydrolysis in the same way as silver salts. The formation of a complex compound via nitrogen electron pair also has a great impact on acidity. It must be assumed that these substances exhibit better chemical stability.

### 2.4. Cytotoxic Activity

**Figure 1:**
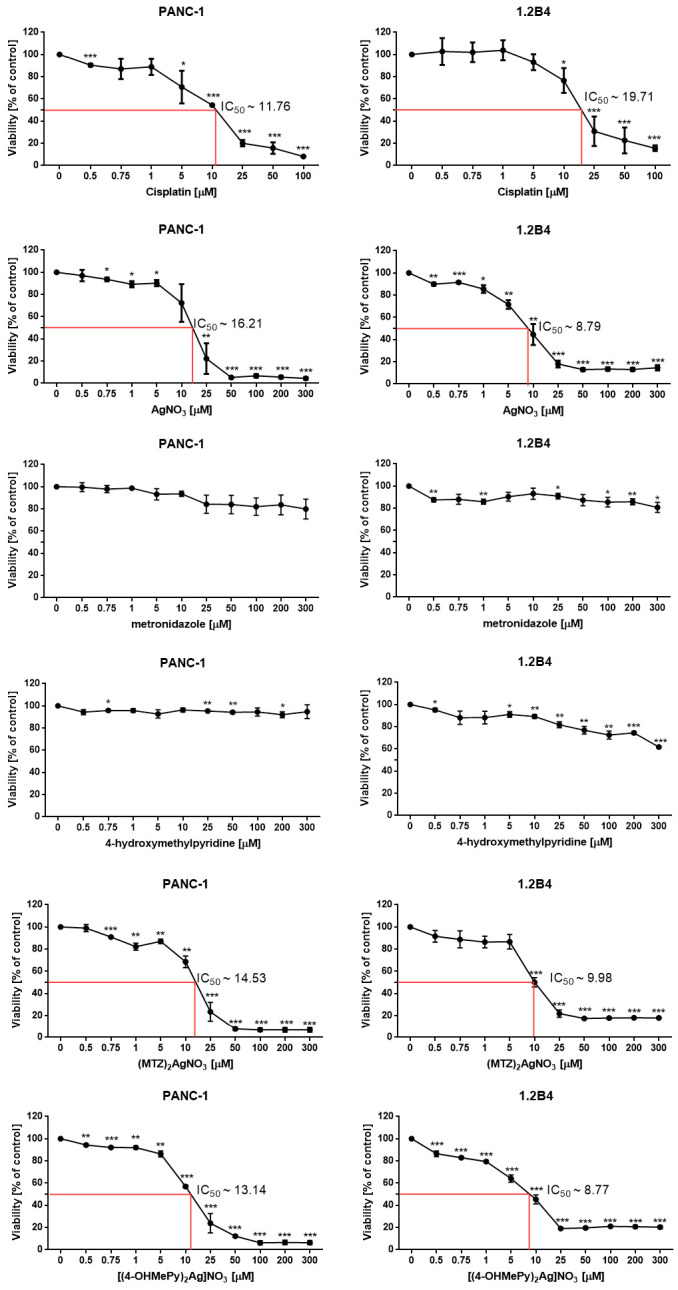
The cytotoxic activity of silver nitrate (AgNO_3_), metronidazole, 4-hydroxymethylpyridine, ((MTZ)_2_Ag)NO_3_, and ((4-OHMePy)_2_Ag)NO_3_ against PANC-1 and 1.2B4 cells. The cells were treated with the tested compounds in the 0–300 µM concentration range, and cisplatin (0–100 µM) was employed as a positive control. After 72-h of incubation, cytotoxicity was determined using the MTT assay. Data are expressed as means ± SD from three to five independent experiments. * *p* < 0.05; ** *p* < 0.01, *** *p* < 0.001 in comparison to untreated cells (negative control).

The cytotoxic action of silver (I) complexes of metronidazole and 4-hydroxymethylpyridine in comparison to silver nitrate, metronidazole, and 4-hydroxymethylpyridine against PANC-1 and 1.2B4 cells is depicted in Figure 1. We found that silver nitrate and its complexes evoked a dose dependent reduction of 1.2B4 and PANC-1 cells viability after 72 h exposure. In the tested concentration range, metronidazole did not reduce the viability of the tested pancreatic cancer cells below 80%. We observed the similar action in PANC-1 cells exposed to 4-hydroxymethylpyridine for 72 h. However, the viability of 1.2B4 cells was not lower than 80% after treatment with 4-hydroxymethylpyridine at concentrations higher than 25 µM. As a positive control, we employed cisplatin that exhibited a dose dependent cytotoxic effect against both human pancreatic cell lines. 

Having compared the cytotoxic potential of the complexes to silver (I) nitrate against the PANC-1 cells, we found that ((MTZ)_2_AgNO_3_) and ((4-OHMePy)_2_Ag)NO_3_ displayed slightly higher cytotoxic activity than silver nitrate (IC50 = 14.53; IC50 = 13.14 vs. IC50 = 16.21, respectively). In the case of 1.2B4 cells, silver (I) ion, both in the form of silver nitrate (IC50 = 8.79) and ((MTZ)_2_AgNO_3_) (IC50 = 9.98) and ((4-OHMePy)_2_Ag)NO_3_ (IC50 = 8.77) complexes exhibited similar cytotoxic potential. The results may suggest that carriers metronidazole and 4-hydroxymethylpyridine did not increase the cytotoxic potential of silver (I) ion in 1.2B4 cells. It should also be noted that the individual IC50 values for silver nitrate and its complexes were lower for 1.2B4 cells than those for PANC-1 cells, suggesting that 1.2B4 cells were more susceptible to silver (I) ions. The data obtained for the positive control revealed that the cytotoxic potential of cisplatin was higher against PANC-1 cells (IC50 = 11.76) in comparison to 1.2B4 cells (IC50 = 19.71). 

In our previous study with the Balb/c 3T3 cell line IC50 values for silver nitrate, 4-hydroxymethylpyridine complex and metronidazole complex with silver nitrate were 2.13, 3.37, 2.19 µM, respectively. For compounds that are free ligands, we had 4-hydroxymethylpyridine and metronidazole at >219 and >227 µM, respectively [9]. Interestingly, we also assessed the percentage of viable murine fibroblasts cells 10T1/2 at concentrations that represented the IC50 for B16 cells (murine melanoma) for silver nitrate and its complexes with 4-hydroxymethylpyridine, as well as metronidazole. The obtained results indicated that the non-cancerous 10T1/2 cells were more resistant to tested compounds than the cancer B16 cells [16].

### 2.5. DNA Damage

**Figure 2:**
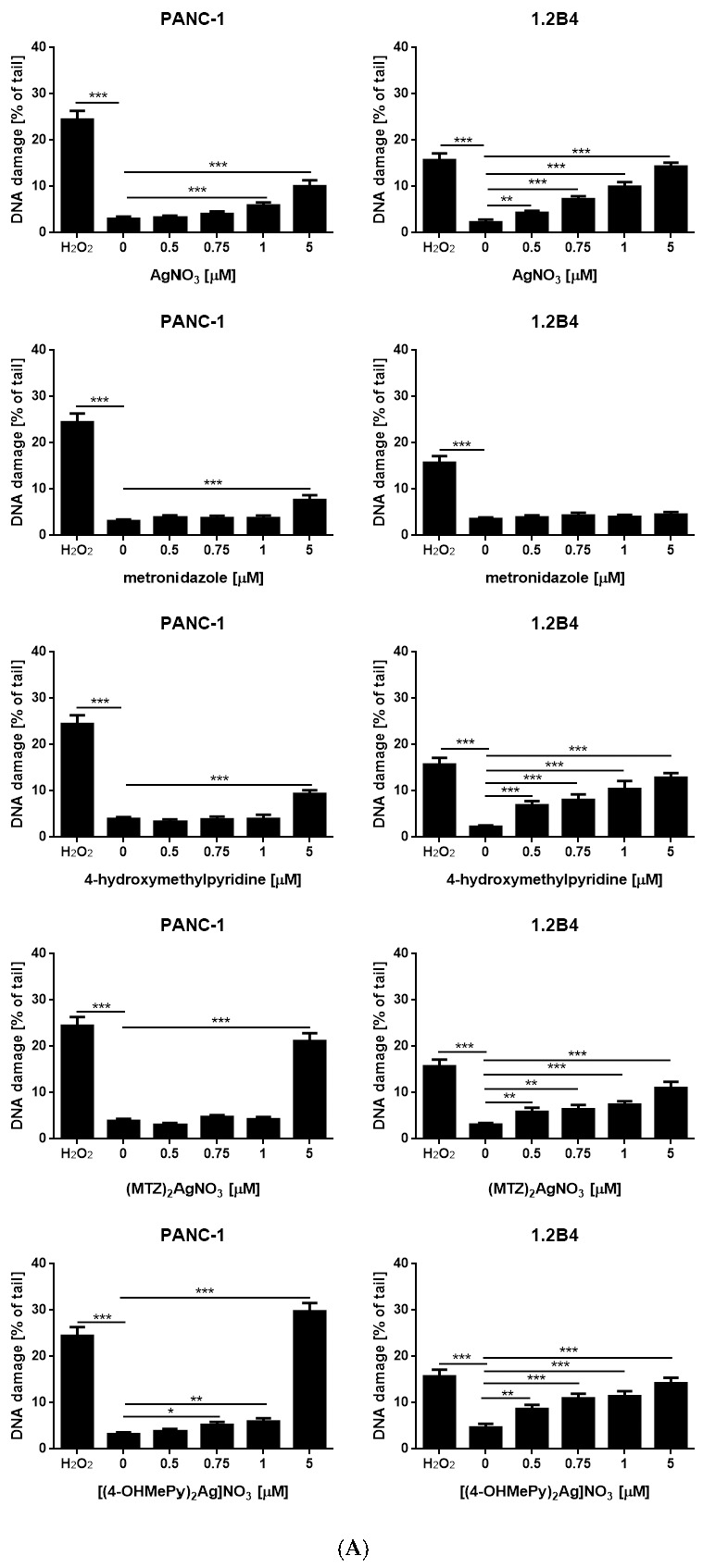

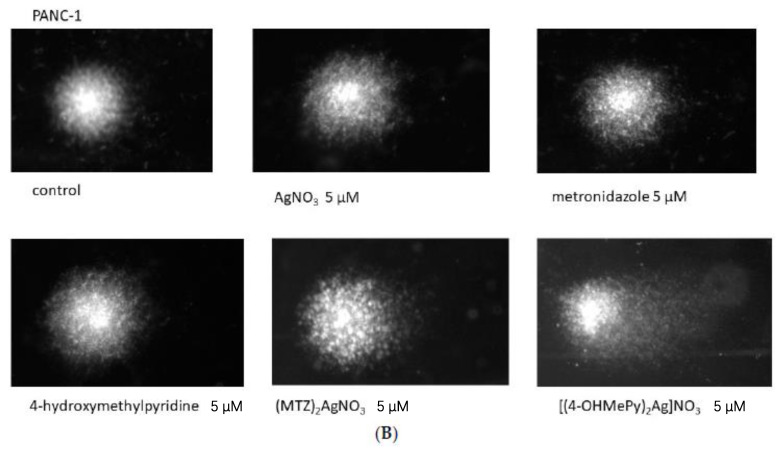

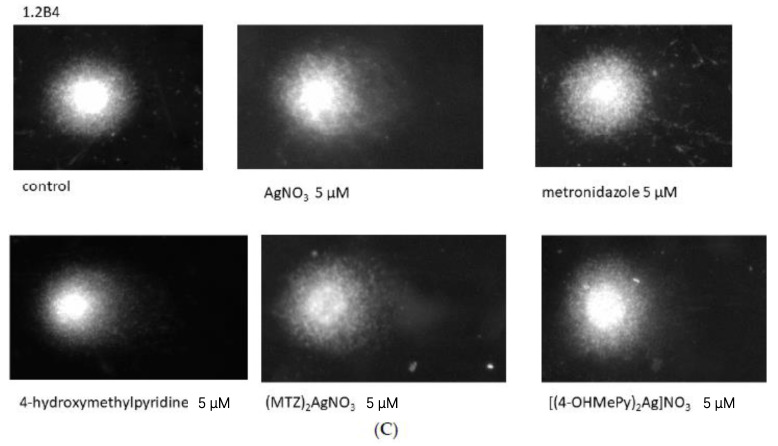
(**A**) The level of DNA damage in PANC-1 and 1.2B4 cells exposed to silver nitrate (AgNO_3_), metronidazole, 4-hydroxymethylpyridine, ((MTZ)_2_Ag)NO_3_, and ((4-OHMePy)_2_Ag)NO_3_ (0.0, 0.5, 0.75, 1.0, 5.0 µM) for 1 h at 37 °C. The DNA damage was determined by the alkaline comet assay. Treatment with 20 µM H_2_O_2_ for 10 min on ice served as a positive control. Data are expressed as mean ± SEM. * *p* < 0.05; ** *p* < 0.01, *** *p* < 0.001 in comparison to untreated cells (negative control). (**B,C**) The representative images of comets after exposure to the tested agents visualized by staining with 2 mg/mL 4′,6-diamidino-2-phenylindole dihydrochloride (DAPI) and observed under fluorescence microscope (Nikon, Tokyo, Japan) at 200× magnification.

The effect of silver (I) complexes of metronidazole and 4-hydroxymethylpyridine in comparison to silver nitrate, metronidazole, and 4-hydroxymethylpyridine on DNA damage of PANC-1 and 1.2B4 cells is presented in Figure 2. We observed that the silver nitrate evoked a dose dependent increase of DNA damage in PANC-1 and 1.2B4 cells. Similarly, exposure of both human pancreatic cancer cells to silver (I) complex of 4-hydroxymethylpyridine induced a dose-dependent increase in DNA damage. This effect was especially visible in 1.2B4 cells although the most distinct DNA damaging effect was noted in PANC-1 cells treated with 5 µM of ((4-OHMePy)_2_Ag)NO_3_. The level of DNA damage in 1.2B4 cells grew as the concentration of silver (I) complex of metronidazole increased. In the case of PANC-1 cells, the treatment with ((MTZ)_2_Ag)NO_3_ did not exert any significant elevation of DNA damage, except for the highest concentration (5 µM). Exposure to the increasing concentration of metronidazole did not elevate the level of DNA damage in both pancreatic cancer cell lines. We only observed a slight increase in DNA damage in PANC-1 cells exposed to 5 µM of metronidazole. In turn, exposure to 4-hydroxymethylpyridine evoked a gradual rise in DNA damage in 1.2 B4 cells, but not in PANC-1 cells. A significant rise in DNA damage in PANC-1 cells was detected after exposure to 5 µM of 4-hydroxymethylpyridine. 

Taken together, the obtained results suggest that silver (I) ion—in the forms of silver nitrate, metronidazole, and 4-hydroxymethylpyridine complexes—shows dose dependent genotoxic properties in human pancreatic cancer cells (Figure 2B,C).

### 2.6. Detection of Apoptosis/Cytometric Analysis

**Figure 3:**
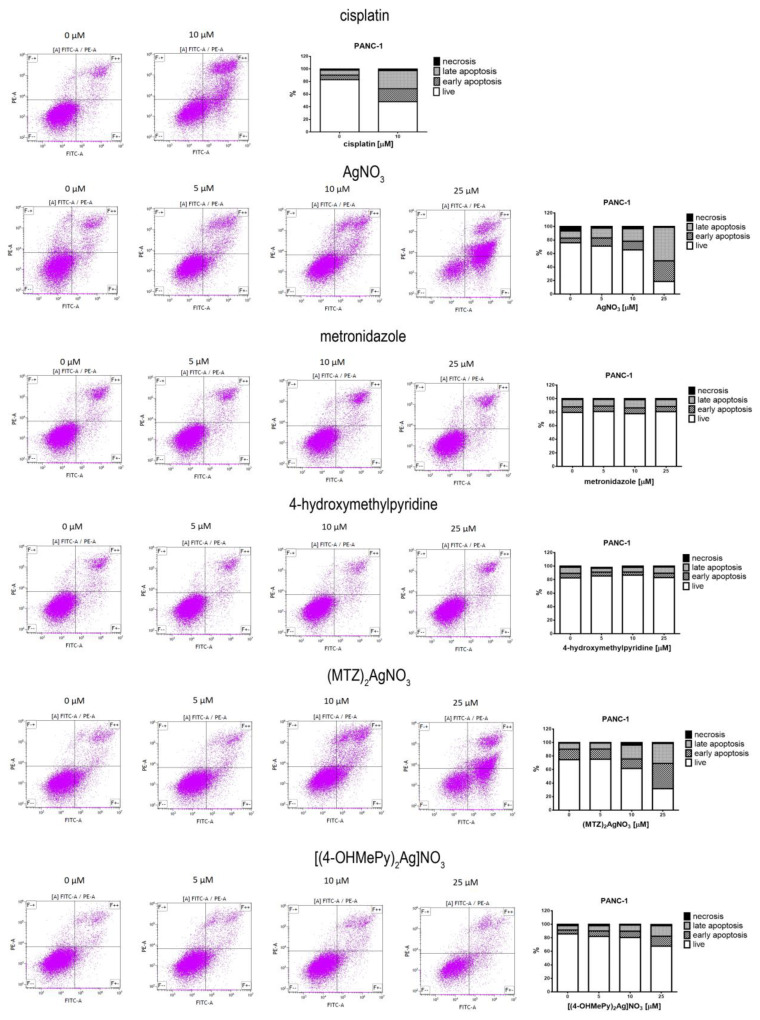

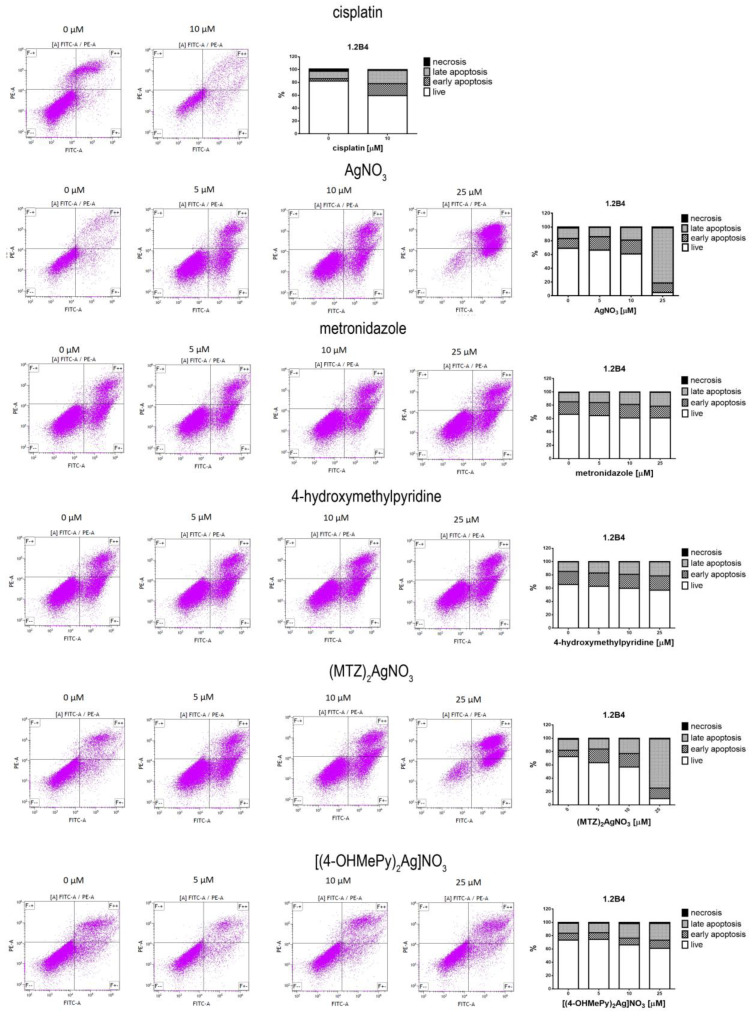
The flow cytometric (Annexin V-FITC/PI) analysis of apoptosis of PANC-1 (**A**) and 1.2B4 (**B**) cells induced by the exposure to silver nitrate (AgNO_3_), metronidazole, 4-hydroxymethylpyridine, ((MTZ)_2_Ag)NO_3_, and ((4-OHMePy)_2_Ag)NO_3_ (0.0, 5, 10, 25 µM) for 72 h. As a positive control 10 µM cisplatin was used. After treatment with the tested compounds, the cells were stained with Annexin V and propidium iodide (PI), left for 15 min in the dark and analyzed by flow cytometer. The percentages of cells in early (Annexin V+, PI−; lower right quadrant; F+– and late apoptotic-necrotic stages (Annexin V+, PI+; upper right quadrant; F++) are shown. The results are representative of three independent experiments.

The potential to induce apoptotic cell death of silver (I) complexes of metronidazole and 4-hydroxymethylpyridine in comparison to silver nitrate, metronidazole, and 4-hydroxymethylpyridine is presented in Figure 3. Based on the results obtained with the MTT assay, we decided to use 5, 10, and 25 µM concentrations of the tested compounds in the Annexin V-FITC/PI flow cytometry. Cisplatin (10 µM) was used as a positive control. We found that silver nitrate and both of its complexes with metronidazole and 4-hydroxymethylpyridine induced a dose-dependent apoptosis of PANC-1 and 1.2B4 cells. What is more, ((MTZ)_2_Ag)NO_3_ had higher proapoptotic activity than silver nitrate and ((4-OHMePy)_2_Ag)NO_3_. Interestingly, silver nitrate was more effective than ((4-OHMePy)_2_Ag)NO_3_. The 72-h exposure to metronidazole and 4-hydroxymethylpyridine did not cause the apoptosis of PANC-1 cells in comparison to untreated cells. In the case of 1.2B4 cells, we observed a slight non-significant increase in the percentage of apoptotic cells after 72-h treatment with metronidazole and 4-hydroxymethylpyridine. Moreover, 10 µM cisplatin evoked the apoptotic death of PANC-1 and 1.2B4 cells.

## 3. Discussion

Silver is a well-known antimicrobial agent, and recent studies reveal its anticancer potential. We aimed to investigate the cytotoxic and genotoxic activity of silver nitrate and its complexes in human pancreatic cancer cell lines, namely PANC-1 and 1.2B4. The cytotoxic effect was determined by the MTT viability assay and the flow cytometry Annexin V-FITC/PI apoptosis assay. In turn, the genotoxic action was evaluated using the alkaline comet assay. Our study revealed that both silver nitrate and its complexes with metronidazole and 4-hydroxymethylpyridine induced a dose-dependent decrease of pancreatic cancer cells viability. In addition, 1.2B4 cells were more susceptible to silver nitrate and its complexes than PANC-1 cells. 

Our observations that silver nitrate exerts cytotoxic effect against different cancer cells are in agreement with other results [19,20]. The cytotoxicity (IC50) of silver nitrate depends on the type of cancer cell line. It was demonstrated that the IC_50_ value for a 72 h exposure to silver nitrate was 5 μM for breast, 6.6 μM for hepatic, 13.5 μM for lung, 35 μM for ovarian, 50 μM for cervical cancer cell lines [9,19,20,21]. We noted that IC50 values for silver nitrate in pancreatic cancer cells were 16.2 for PANC-1 μM and 8.8 μM for 1.2B4.

To the best of our knowledge, this is the first study presenting cytotoxicity of silver (I) complexes of metronidazole and 4-hydroxymethylpyridine against pancreatic cancer cells. We found the IC50 values for a 72 h exposure to ((MTZ)_2_Ag)NO_3_ were ~14.5 μM for PANC-1 and ~10.0 μM for 1.2B4 cells. In turn, the IC50 values for a 72-h exposure to ((4-OHMePy)_2_Ag)NO_3_ were ~13.1 μM for PANC-1 and ~8.8 μM for 1.2B4 cells. Our previous study employed silver (I) complexes of metronidazole and 4-hydroxymethylpyridine against Balb/c 3T3 and HepG2, showing that IC50 were ~2.2 μM and ~7.6 μM for ((MTZ)_2_Ag)NO_3_ and ~3.4 μM and ~6.5 μM for ((4-OHMePy)_2_Ag)NO_3_, respectively [9]. Taken together, our observation supports cytotoxic and anticancer properties of silver nitrate and its metronidazole and 4-hydroxymethylpyridine complexes against various cancer cells, including pancreatic cancer cells. The molecular background of this anticancer action is suggested to be associated with the induction of apoptosis [10,22,23,24]. The results obtained in this study also support this hypothesis, since we demonstrated that silver nitrate and its complexes with metronidazole and 4-hydroxymethylpyridine induced a dose-dependent apoptotic death of PANC-1 and 1.2B4 cells. Evidence indicated that apoptosis of different types of cancer cells evoked by silver (I) complexes was mediated by oxidative stress [25,26,27]. Recently, Altay et al. demonstrated that silver (I) complexes induced oxidative stress via cellular generation of reactive oxygen species (ROS), especially hydroxyl radicals, in MCF7 cells [28]. Li et al. revealed that silver nitrate generated more ROS in TK6 cells than silver nanoparticles [8]. In turn, in NIH3T3 cells, apoptotic effect of silver (I) complexes was found to be mediated by mitochondrial ROS generation and JNK activation [27].

The obtained results showed that metronidazole and 4-hydroxymethylpyridine were not cytotoxic against pancreatic cancer cells. These results are in line with our previous observation in Balb/c 3T3 and HepG2 cells [9] and B16 and 10T1/2 cell lines [16]. In both cell lines, used concentration range of metronidazole and 4-hydroxymethylpyridine did not significantly affect the viability of PANC-1 and 1.2B4 cells which was mostly greater than 50%. The flow cytometric analysis also revealed that metronidazole and 4-hydroxymethylpyridine did not induce apoptosis of the studied pancreatic cancer cells.

Due to the fact that DNA damage is a major inducer of apoptotic cell death, we decided to determine the level of DNA damage induced by silver nitrate and its complexes with metronidazole and 4-hydroxymethylpyridine. We found that silver nitrate evoked a dose-dependent increase of DNA damage in PANC-1 and 1.2B4 cells. The observation stays in line with the results obtained by Li et al. who, using the micronucleus assay, reported that silver nitrate significantly increased the number of micronucleus frequency in TK6 cells. What is more, the co-administration of silver nitrate with N-acetyl cysteine and trolox (a well-known ROS scavenger) decreased the number of micronucleus frequency, supporting the role of oxidative stress in silver (I) mediated DNA damage [11]. The dose dependent rise in DNA damage was detected after exposure to ((4-OHMePy)_2_Ag)NO_3_ in both pancreatic cell lines. Moreover, 4-hydroxymethylpyridine evoked a dose dependent increase in DNA damage in 1.2B4 cells, whereas, in PANC-1 cells, only the highest concentration caused a significant elevation of DNA damage. In turn, the genotoxicity of ((MTZ)_2_Ag)NO_3_ and metronidazole against 1.2B4 cells was dose dependent. In case of PANC-1 cells, ((MTZ)_2_Ag)NO_3_ and metronidazole displayed DNA damaging effect only at the highest concertation (5 μM). To conclude, it seems that 1.2B4 cells showed greater sensitivity to genotoxic action of silver nitrate and its complexes.

Complex compounds showed greater stability than AgNO_3_, namely their degradation progressed slower after exposure to daylight. The obtained results indicate that the silver (I) complexes of metronidazole and 4-hydroxymethylpyridine may have a potential application in pharmacy since their aqueous solutions showed greater stability when exposed to both daylight and pure UV-A radiation. The decomposition of the compounds only occurred after evaporation of the solvent. Moreover, the Ag(I) complexes of metronidazole and 4-hydroxymethylpyridine depicted desirable properties, such as good solubility in water, thermal stability up to 80 °C, pronounced antimicrobial activity against both Gram-positive and Gram-negative bacteria, including *Escherichia coli*, *Klebsiella pneumoniae*, and *Candida albicans*. Recently, we demonstrated that silver (I) complex of metronidazole was efficiently used in the treatment of Ocular Rosacea; thus, it may be an alternative method of acne rosacea therapy [29].

## 4. Materials and Methods 

### 4.1. Chemicals and Reagents

The synthesis of complex compounds of silver nitrate with metronidazole (MTZ) and with 4-hydroxymethylpyridine (4-OHMePy) was carried out as described in our previous publications [9]. We used solvents of reagent purity. Analytical standards of metronidazole (CAS: 443-48-1; M.W. 171.5 g/mol), 4-hydroxymethylpyridine (CAS: 586-95-8; molecular weight: 110 g/mol), and silver nitrate (CAS: 7761-88-8; molecular weight: 169 g/mol) were purchased from Sigma-Aldrich (Poznań, Poland). The RPMI 1640 medium, fetal calf serum (FCS), Trypsin–EDTA solution, and penicillin/streptomycin solution were purchased from Gibco (Life Technologies, Carlsbad, CA, USA). Dulbecco’s modified Eagle’s medium (DMEM) and fetal bovine serum were supplied by American Type Culture Collection (ATCC) (Rockville, MD, USA). All other chemicals were purchased from commercial suppliers and were of the highest available purity. Melting points were determined with a Boethius apparatus (Franz Küstner Nachf. KG, HMK, Dresden, Germany) and have been uncorrected. Elemental analysis was performed using the Perkin Elmer 2400 Series II CHNS/O Analyzer (PerkinElmer, Waltham, MA, USA). A lamp with a wavelength of 365 nm was used as the source of UV-A rays (Vilber Lourmat, Marne-la-Valée, France). We determined the pH of aqueous solutions of the complex compounds using a Hanna Instruments HI 221 pH-meter (Hanna Instruments SRL, Nagyfalu, Romania).

### 4.2. Synthesis

#### 4.2.1. Synthesis of Metronidazole Complex with Silver Nitrate

We described synthesis earlier [9]. Exactly 1.71 g (10 mmol) of metronidazole was added into the solution of silver nitrate (0.85 g, 5 mmol) in 25 mL of water. We stirred the reaction mixture at 70–80 °C temperature for 2 min. After cooling, the compound was crystallized from the aqueous solution, purified, and dried. The results were confirmed and in line with our previous publications [29] (molecular weight: 512.182 g/mol; yield: 2.3 g (89.84%)). Elemental analysis measured (calc. %): C 28.32 (28.14), H 3.12 (3.54), N 19.13 (19.14); melting point: 151–152 °C. ESI-MS (methanol) *m/z*: 449.035 ((MTZ)_2_Ag)^+^. ^1^HNMR spectrum is shown in Supplementary Materials as Figure S4.

#### 4.2.2. Synthesis of 4-hydroxymethylpyridine Complex with Silver Nitrate

Synthesis was based on the procedure described by us earlier [9] wherein 1.36 g (8.0 mM) of silver nitrate was added into a solution of 4-hydroxymethylpyridine (1.75 g, 16.0 mM) in 30 mL of water. The reaction mixture was mixed intensively at room temperature for 4 h. Impurities from the mixture in gray color were filtered off. The solution was evaporated producing a slightly light-yellow precipitate. We purified the crude product by recrystallization in ethanol obtaining slightly yellow crystals. Molecular weight 388.126 g/mol; yield: 2.55 g (81.99%). Elemental analysis measured (calc. %): C 37.21 (37.13), H 3.58 (3.64), N 10.93 (10.83); melting point: 128–129 °C. ESI-MS (methanol) *m/z*: 325.01 ((4-OHMePy)_2_Ag)^+^. ^1^HNMR spectrum is shown in Supplementary Materials as Figure S5.

### 4.3. Preparation of the Solutions for Tests

The compounds (complexes) were dissolved in water to obtain a 0.01 M stock solution. Comparative solutions were AgNO_3_, MTZ, and 4-OHMePy, which were prepared in the same way. A 0.005 M (5000 µM) stock aqueous solution was used to prepare the cisplatin solution with the concentrations of 0.5, 0.75, 1, 5, 10, 25, 50, and 100 µM.

### 4.4. Light Stability of the Tested Compounds

Light stability of the complex compounds ((MTZ)_2_Ag)NO_3_ and ((4-OHMePy)_2_Ag)NO_3_, as well as the silver nitrate, was tested in direct daylight in the air and in the dark. The complexes were dissolved in water; the concentration of each solution was 0.06 M. This concentration corresponds to the concentration usually used in medicine, i.e., 1% silver nitrate [30] and 2% metronidazole. Next, 3 µmol of the complexes and silver salt were the reference system (50 µL of the prepared 0.06 M solutions) and applied onto various groundworks: tissue paper, paper, glass, and leather imitation. The stability of the complexes and silver nitrate was visually monitored for 108 h, making photography documentation after 0, 1, 4, 18, 24, 40, 48, 52, 60, 84, and 108 h. Moreover, the rate of decomposition of silver (I) complexes with metronidazole and 4-hydroxymethylpyridine by UV-A radiation was assessed. The test substrate was tissue paper. For comparison, silver (I) nitrate and ligand solutions (metronidazole and 4-hydroxymethylpyridine) were also used. Panels with images of visual changes from 0 to 1800 s recorded after exposure of the compounds to UV-A radiation (0.06 M concentration, 50 µL of the solution) are shown in Supplementary Materials as Figures S1–S3.

The pH of 0.06 M aqueous solution of the metronidazole complex was determined using a pH-meter and was 4.9–5.1. The pH of 0.06 M aqueous solution of the 4-hydroxymethylpyridine complex was determined using a pH-meter and was 6.3–6.5. Comparative 0.06 M aqueous solutions, on the other hand, showed the following pH values: 4.1–4.2 (silver nitrate); 4.5–4.7 (metronidazole); 5.5–5.7 (4-hydroxymethylpyridine).

### 4.5. Cell Culture

Next, the 1.2B4 cell line (human hybrid cell line that was formed by fusion of primary culture of human pancreatic islets with human pancreatic carcinoma cell line (HuP-T3)) was purchased from European Collection of Authenticated Cell Cultures (ECACC, Salisbury, UK). The PANC-1 cancer cell line was bought from the American Type Culture Collection (ATCC, Manassas, VA, USA). All cell lines were grown as a monolayer in standard conditions: 37 °C, 100% humidity, and the atmosphere of 5% CO_2_ and 95% air. Furtherm 1.2B4 cells were cultured in RPMI 1640 medium containing 2 mM glutamine supplemented with 10% fetal calf serum and 50 IU/mL penicillin/streptomycin. PANC-1 cell line was grown in DMEM supplemented with 10% fetal bovine serum, and 50 IU/mL penicillin/streptomycin. Both cell lines grow in monolayer and trypsin-EDTA solution was used to detach. Moreover, 1.2B4 cells, after the third to fifth passage, were used for the experiments. The trypan blue staining was employed to count the number of the living cells.

### 4.6. Cytotoxicity Evaluation: MTT Assay

The cytotoxicity of silver nitrate (AgNO_3_), metronidazole, 4-hydroxymethylpyridine, ((MTZ)_2_Ag)NO_3_, and ((4-OHMePy)_2_Ag)NO_3_ against PANC-1 and 1.2B4 cells was determined using MTT (3-(4,5 dimethyl-thiazol-2-yl)-2, 5-diphenyl tetrazolium bromide) assay. Next, 1.2B4 cells were seeded in 96-well plates at a density of 2000 cells per well and incubated to grow overnight to achieve logarithmic growth phase. Next, the studied compounds (0.5, 0.75, 1, 5, 10, 25 50, 100, 200, and 300 µM) were added to the cells and cultured for 72 h. As a positive control, we exposed the studied cell lines to 0.5, 0.75, 1, 5, 10, 25, 50, and 100 µM of cisplatin for 72 h. After 72 h of incubation, 10 µL of MTT solution (5 g/L) was added into each well and incubated for additional 4 h. Subsequently, the medium was removed and 100 µL of dimethyl sulfoxide (DMSO) was added into each well. DMSO dissolves formazan crystals. Then, the absorbance was measured at a wavelength of 570 nm by using a microplate reader (SpectrostarNano, BMG Biotech, (BMG Biotech Inc., Cary, NC, USA). Mean value from three to five independent PANC-1 and 1.2B4 cell cultures was taken to calculate cell viability expressed as a percentage of control (%). The control group (untreated cells) was considered as 100%.

### 4.7. Detection of Apoptosis

Apoptosis detection and quantification were analyzed using the Annexin V and FITC kit (BD Biosciences, San Diego, CA, USA), as per the manufacturer’s protocol and instructions. PANC-1 and 1.2B4 cells were trypsinized and seeded at a density of 0.5 × 10^6^ cells in each well in a 6-well plate and incubated to grow overnight to achieve logarithmic growth phase. Next, the cells were washed with PBS, followed by the addition of a fresh medium and different concentrations (5, 10, 25 μM) of silver nitrate and its complexes with metronidazole and 4-hydroxymethylpyridine, metronidazole, and 4-hydroxymethylpyridine. Next, 10 µM cisplatin was employed as a positive control. After 72 h, the cells were trypsinized, washed with PBS twice after exposure, collected by centrifugation, and then resuspended in a flow cytometry binding buffer. Moreover, 100µL of cell suspension containing 10^5^ cells was left in the dark with 5 µL of the apoptosis detection kit (Annexin V-FITC/PI, BD Biosciences, San Diego, CA, USA) for 15 min at room temperature. Then, the induction of apoptosis was determined using a flow cytometer (FACSCalibur, BD Biosciences, San Diego, CA, USA). Each material was tested in triplicate and three independent experiments were performed.

### 4.8. DNA Damage Determination: Alkaline Comet Assay

The alkaline version of the comet assay was performed according to the protocol of Singh et al. [31] with previously described modifications [32,33]. This comet assay version detects single and double strand breaks as well as the alkaline-labile sites, which are considered endogenous DNA damage. The cells were exposed to silver nitrate (AgNO_3_), metronidazole, 4-hydroxymethylpyridine, ((MTZ)_2_Ag)NO_3_, and ((4-OHMePy)_2_Ag)NO_3_ at the concentration range 0, 0.5, 0.75, 1, and 5 µM for 1 h at 37 °C. As a positive control, the cells were exposed to H_2_O_2_ (20 µM) for 10 min on ice. Then, the cells were washed two times with cold PBS, suspended in low melting point agarose (0.75%), and spread onto normal melting point agarose (0.5%) precoated microscope slides. The cells were then lysed by incubation in a lysis buffer (NaCl, 2.5 M; EDTA, 100 mM; TritonX100, 1%; and Tris, 10 mM; pH 10) at 4 °C for 1 h. Next, the slides were suspended in unwinding buffer (NaOH, 300 mM; EDTA, 1 mM; pH > 13) and electrophoresis was carried out at 0.73 V/cm (28 mA) for 20 min. After electrophoresis, the slides were washed three times with distilled water, drained, and stained with 2 mg/mL 4′,6-diamidino-2-phenylindole dihydrochloride (DAPI) under dark conditions at 4 °C for 30 min. Finally, the comets were observed at 200× magnification under a fluorescence microscope (Nikon, Tokyo, Japan) connected to a video camera with ultraviolet (UV1), filter block and personal computer equipped with the LuciaComet v. 4.51 analysis software (Laboratory Imaging, Prague, Czech Republic). DNA damage as the percentage of DNA in the tail of the observed comet was evaluated from 50 cells in each sample.

### 4.9. Data Analysis

The results of the MTT assay and flow cytometry are expressed as mean ± SD. In the case of the data derived from the comet assay, the results are presented as mean and standard error of the mean (±SEM). One-way analysis of variance (ANOVA) was applied. The 50% inhibition (cytotoxicity) concentrations (IC50) were derived from the dose-dependent curve plotted with the concentration of the compound vs. percentage of viability. The IC50 values were calculated by the GraphPad Prism 6.0 software (GraphPad, San Diego, CA, USA); *p* value less than 0.05 was considered as statistically significant. All statistical analysis were performed using GraphPad Prism 6.0. 

## 5. Conclusions

Our findings suggest that silver nitrate and its complexes with metronidazole and 4-hydroxymethylpyridine exhibit proapoptotic and genotoxic properties against human pancreatic cancer cells. We observed that 1.2 B4 cells were more sensitive to the tested agents than PANC-1 cells.

The silver (I) complex of formula ((4-OHMePy)_2_Ag)NO_3_ was less stable than ((MTZ)_2_Ag)NO_3_. Daylight significantly affected the rate of silver salt (nitrate) decomposition. 

A combination of six properties of Ag(I) complexes of metronidazole and 4-hydroxymethylpyridine were found: (i) good light stability, (ii) good water solubility, (iii) good thermal stability, (iv) no toxicity of the organic ligand (metronidazole and 4-hydroxymethylpyridine), (v) antimicrobial activity, and (vi) antitumor activity in vitro, all of which make them good candidates for medical applications.

## Supplementary Materials

The following are available online at https://www.mdpi.com/2072-6694/12/12/3848/s1, Figure S1. Groundworks with applied solutions of: silver nitrate (A) and complexes of silver: with metronidazole (B) and 4- hydroxymethylpyridine (C) left in the dark and photographed after the time shown. Figure S2. Groundworks with applied solutions of: silver nitrate (A) and complexes of silver: with metronidazole (B) and 4- hydroxymethylpyridine (C) left in the light and photographed after the time shown. Figure S3: Tissue-paper with applied solutions of silver nitrate, metronidazole, 4-hydroxypyridine, and complexes of silver with metronidazole and 4-hydroxymethylpyridine left in UV-A and photographed after the time shown; Figure S4: ^1^H NMR (600MHz, DMSO) spectra of metronidazole (A) and silver (I) complex with metronidazole (B); Figure S5: ^1^H NMR (600MHz, DMSO) spectra of 4-hydroxymethylpyridine (A) and silver (I) complex with 4-hydroxymethylpyridine (B).
