# Supplementary material for: Light Stability, Pro-Apoptotic and Genotoxic Properties of Silver (I) Complexes of Metronidazole and 4-Hydroxymethylpyridine against Pancreatic Cancer Cells In Vitro
Source: Cancers (Basel). 2020 Dec 20;12(12):3848. doi: 10.3390/cancers12123848 (PMC7767315; doi:10.3390/cancers12123848)
Supplement: Supplementary file 1 [file cancers-12-03848-s001.pdf]

Supplementary Materials

Light Stability, Pro-Apoptotic and Genotoxic Properties of Silver(I) Complexes with Metronidazole and 4-hydroxymethylpyridine against Pancreatic Cancer Cells In Vitro

Dominik Żyro, Agnieszka Śliwińska, Izabela Szymczak-Pajor, Małgorzata Stręk and Justyn Ochocki

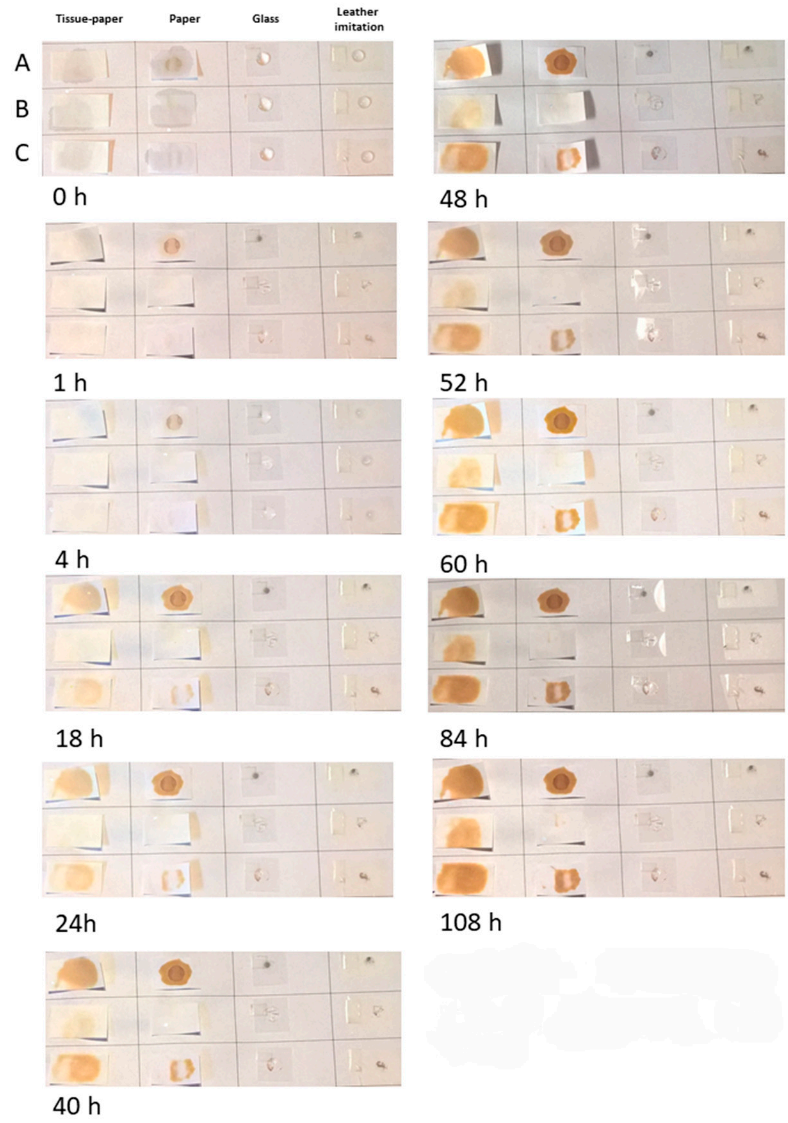

Figure S1. Groundworks with applied solutions of: silver nitrate (**A**) and complexes of silver: with metronidazole (**B**) and 4-hydroxymethylpyridine (**C**) left in the dark and photographed after the time shown.

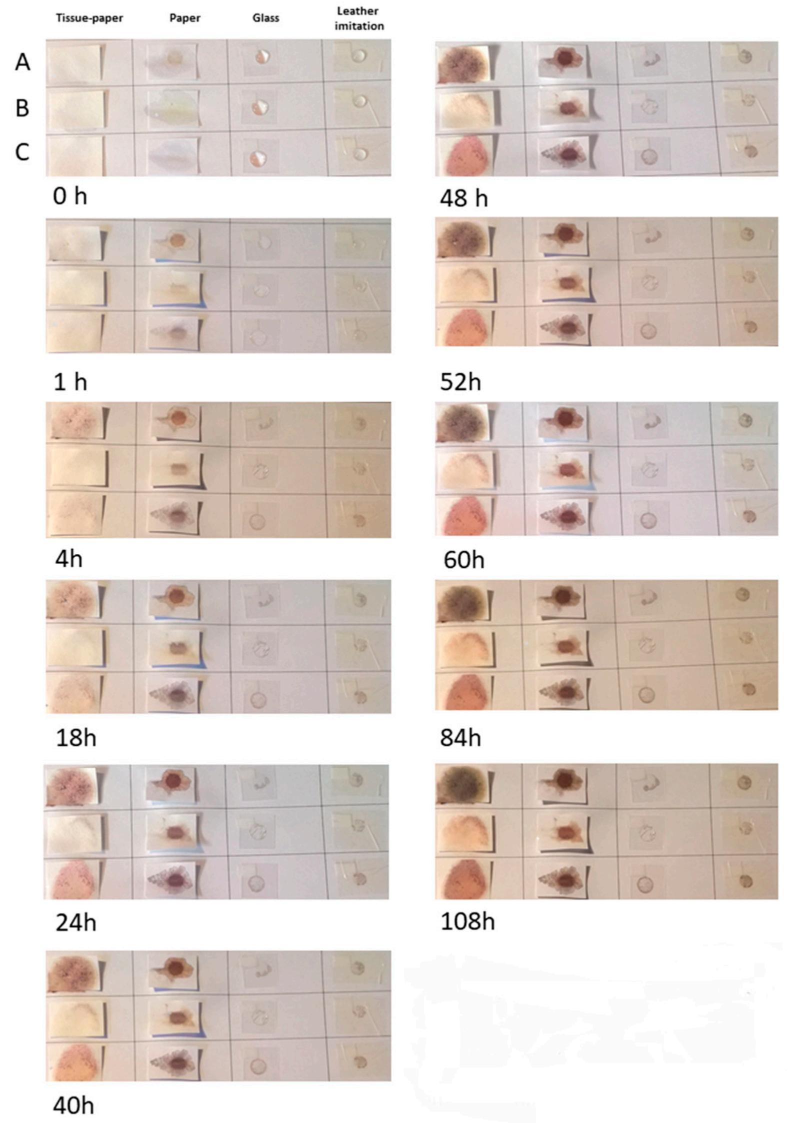

Figure S2. Groundworks with applied solutions of: silver nitrate (**A**) and complexes of silver: with metronidazole (**B**) and 4-hydroxymethylpyridine (**C**) left in the light and photographed after the time shown.

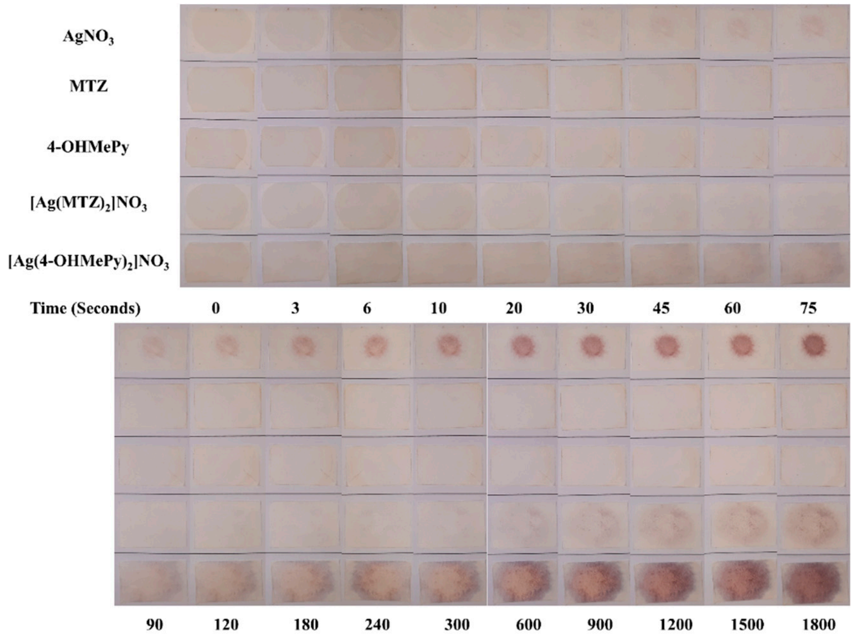

Figure S3. Tissue-paper with applied solutions of: silver nitrate, metronidazole, 4-hydroxypyridine and complexes of silver: with metronidazole and 4-hydroxymethylpyridine left in UV-A and photographed after the time shown.

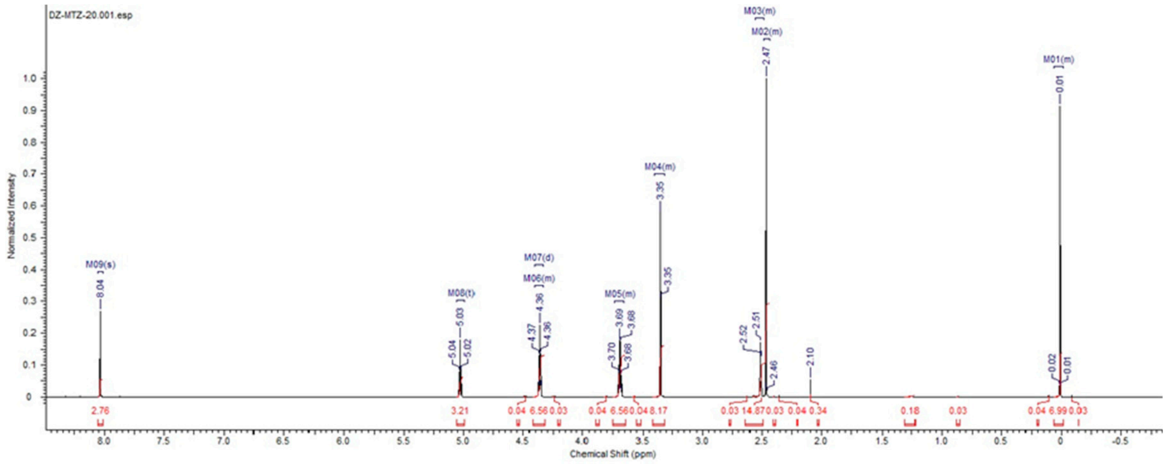

(A)

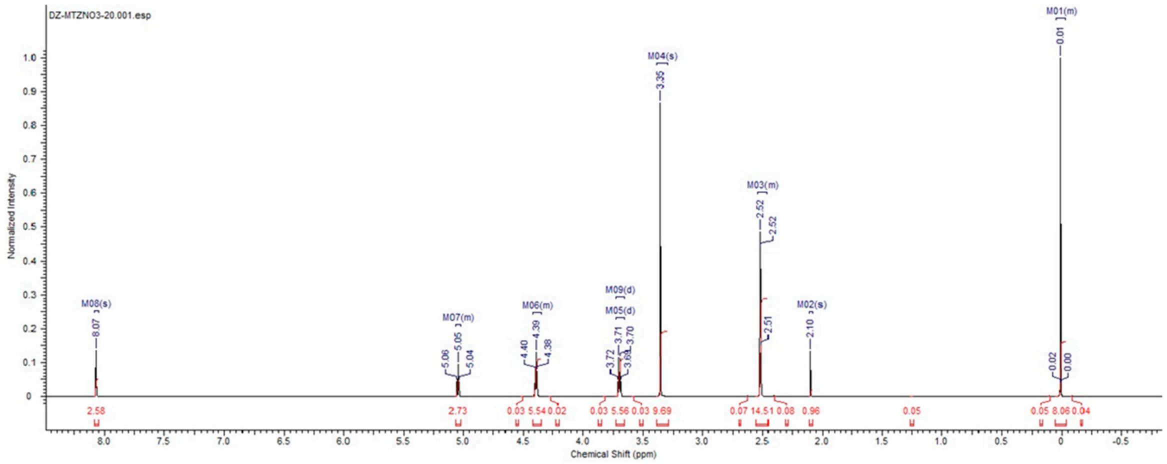

(B)

Figure S4. ^{1}H NMR (600MHz, DMSO) spectra of metronidazole (**A**) and silver(I) complex with metronidazole (**B**).

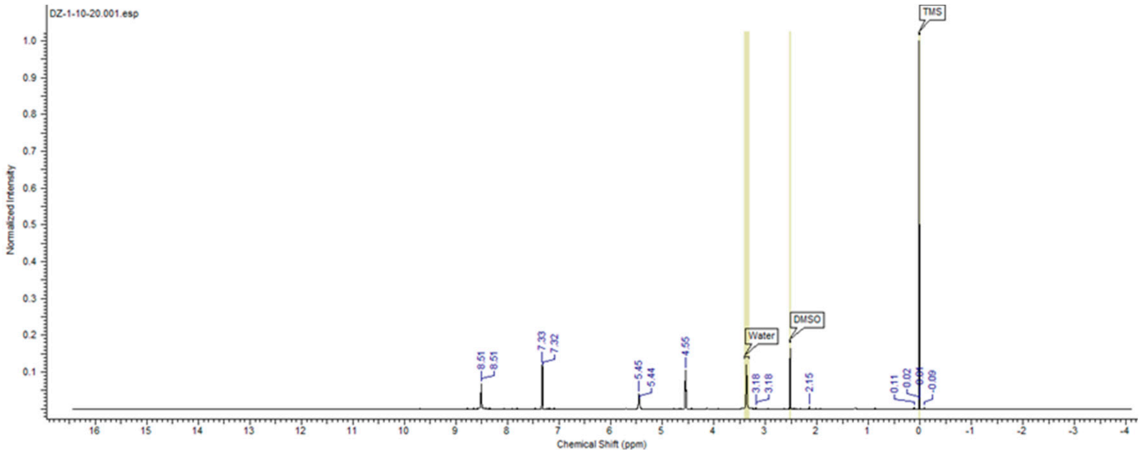

(A)

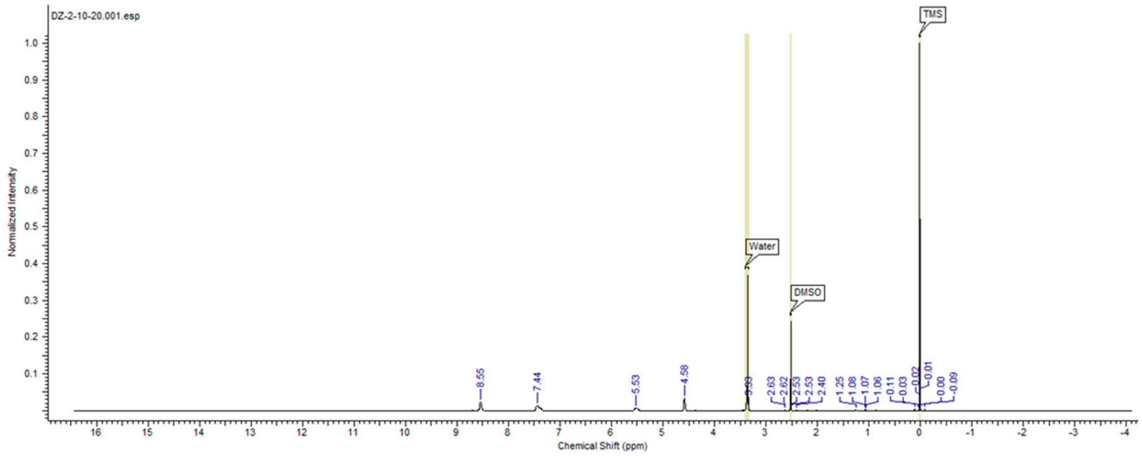

(B)

Figure S5. ^{1}H NMR (600MHz, DMSO) spectra of 4-hydroxymethylpyridine (**A**) and silver(I) complex with 4-hydroxymethylpyridine (**B**).

Publisher's Note: MDPI stays neutral with regard to jurisdictional claims in published maps and institutional affiliations.

© 2020 by the authors. Licensee MDPI, Basel, Switzerland. This article is an open access article distributed under the terms and conditions of the Creative Commons Attribution (CC BY) license (http://creativecommons.org/licenses/by/4.0/).
